# Health Behaviors and Psychological Well-Being Among First-Year Psychology, Medicine, and Nursing Students: A Cross-Sectional Analysis

**DOI:** 10.3390/healthcare13172162

**Published:** 2025-08-30

**Authors:** Natacha Palenzuela-Luis, Gonzalo Duarte-Clíments, Juan Gómez-Salgado, José Ángel Rodríguez-Gómez, María Begoña Sánchez-Gómez

**Affiliations:** 1 University Hospital of Canarias, 38320 San Cristóbal de La Laguna, Spain; 2 Cátedra de Enfermería, University of La Laguna, 38200 San Cristóbal de La Laguna, Spain; 3 Cieza Este Health Centre, Murcia Health Service, 30530 Murcia, Spain; 4 Faculty of Health Sciences, International University of Valencia (VIU), 46002 Valencia, Spain; 5 Department of Sociology, Social Work and Public Health, Faculty of Labour Sciences, University of Huelva, 21007 Huelva, Spain; 6 Safety and Health Postgraduate Program, Universidad Espíritu Santo, Guayaquil 092301, Ecuador; 7 Department of Nursing, University of La Laguna, 38200 San Cristóbal de La Laguna, Spain; 8 Research in Health and Social Care Sciences Program, Catholic University of Murcia (UCAM), 30107 Murcia, Spain

**Keywords:** self-concept, self-perception, physical activity, behavior, lifestyle, psychology, medicine, nursing, students, public health, health promotion, well-being

## Abstract

**Introduction:** Understanding adolescent maturational development and its impact on physical and psychological well-being is essential for supporting the academic and professional growth of undergraduate students in Health Sciences programs (Psychology, Medicine, and Nursing). This study aimed to assess and compare self-concept, self-perception, physical activity, and lifestyle among first-year Health Sciences students. **Methods:** A descriptive cross-sectional study was conducted with first-year students at the University of La Laguna, Tenerife, Spain. Data were collected using the Rosenberg Self-Esteem Scale (RSES), General Health Questionnaire (GHQ-12), Physical Activity Questionnaire for Adolescents (PAQ-A), and Health Behaviour in School-aged Children (HBSC). Variables included sex, age, study program, and body mass index (BMI). Statistical analyses included descriptive statistics, reliability assessment (Cronbach’s alpha), distribution tests, and chi-squared tests. **Results:** Among 190 participants, the RSES showed generally positive self-esteem, although 75% of students reported low self-confidence. Male Psychology students all scored in the fair range on self-perception. Physical activity was low, particularly among female students, with 20% classified as sedentary. HBSC results indicated the need for lifestyle improvements. SOC-13 scores showed that 80.5% of students had fair levels of sense of coherence. **Conclusions:** Health Sciences students exhibited low self-concept, emotional distress, sedentary habits, and inadequate lifestyle behaviors. Male Nursing students and female Psychology students had the poorest self-concept scores. The findings emphasize the need for interventions promoting healthy habits and emotional well-being among students entering health-related academic programs.

## 1. Introduction

### 1.1. Background

Currently, many adolescents experience inadequate health conditions related to self-concept, self-perception, physical activity, and lifestyle [1]. Life satisfaction is associated with lower incidence of pathologies, increased happiness, and improved emotional well-being [1]. Stress directly affects psychological health and contributes to a higher prevalence of cardiovascular disease, cancer, and suppression of the immune system; these effects are often compounded by anxiety and depression [2,3]. All the aforementioned variables provide a holistic understanding of adolescents, encompassing their thoughts, behaviors, emotions, and social perceptions, which in turn influence their interactions with others. Late adolescence, defined as the period between 19 and 24 years of age, is a critical stage in which individuals prepare for adulthood and acquire the skills necessary for career development. This period is considered key for preventive interventions and the promotion of healthy lifestyles [4].

A previous study conducted by the same research team in the Canary Islands (Spain) focused on school-aged children with a mean age of 17 years. The findings indicated that females were more concerned about their health but were less physically active than males. In this population, self-concept and self-esteem were not significantly related to physical activity. Notably, although the Canary Islands have a higher prevalence of obesity than the national average, participants generally reported positive health perceptions and engaged in what appeared to be adequate levels of physical activity. These results suggest that information about healthy habits provided to this age group does not necessarily translate into actual behavioral changes, possibly due to factors such as lack of knowledge, motivation, or resources [4].

Positive adolescent self-concept is indicative of emotional well-being and social integration; a stronger self-concept is associated with higher personal satisfaction [5,6]. Self-concept refers to an individual’s perception of themselves and encompasses physical, cognitive, behavioral, affective, and social dimensions. It is influenced by values, cultural expectations, and personal relationships [7,8,9,10]. Examining self-concept in adolescents is crucial because it serves as a key psychological indicator of emotional well-being and social integration. However, self-concept varies according to age and sex, changing throughout maturational development [5,11].

Self-esteem is closely related to self-concept and is understood as self-respect, self-acceptance, personal worth, or self-efficacy [11,12,13,14]. It reflects how individuals define themselves and serves as an indicator of general well-being [15,16]. Adequate self-esteem can prevent anxiety, depression, and suicidal ideation [13,16,17], whereas low self-esteem is associated with substance use [15] and maladaptive coping strategies [18,19,20,21,22,23,24]. Together, self-concept and self-esteem shape the development of identity and influence how adolescents think, behave, and relate to others [8,12,14].

Self-perception consists of the internally conscious and organized components of self-concept [25,26]. It is influenced by factors such as physical activity, socioeconomic status, and sex. According to the World Health Organization (WHO), more than 300 million people worldwide suffer from anxiety and depression. Accordingly, numerous studies highlight the importance of improving adolescents’ self-perception and promoting physical activity as strategies to enhance mental and physical well-being [27,28,29,30,31,32,33,34,35,36,37].

Physical activity positively influences adolescents’ self-concept, self-esteem, and perceived quality of life by promoting overall development. It is essential for preventing overweight and obesity [28,29], enhancing cognitive functioning, supporting proper physiological processes, and contributing to overall well-being [30,31,32,33]. A positive physical self-concept promotes social integration, autonomy, self-esteem, and healthier lifestyle choices, including reduced consumption of harmful substances [34,35,36,37]. Despite these benefits, physical activity levels remain low, particularly among females [32,33]. This issue is not limited to Spain; consequently, many countries have integrated strategies to promote physical activity into public health policies [30,38]. The World Health Organization (WHO) also establishes age-specific recommendations regarding the duration, intensity, and frequency of physical activity [39,40].

Lifestyle refers to the set of behaviors and daily habits that directly affect an individual’s health, either positively or negatively [10,31]. Maintaining a healthy lifestyle, including regular physical activity, helps prevent eating disorders and chronic non-communicable diseases while improving self-concept, self-esteem, and self-perception [10,31,35,39,41,42,43]. Nevertheless, in Spain, 15.5% of female adolescents and 16.5% of male adolescents are overweight or obese. Additionally, an increasing number of adolescents experience eating disorders, malnutrition, or underweight issues [31,44]. Assessing the habits of young people is therefore critical for understanding and promoting effective health management.

### 1.2. Present Study

The concepts described above, together with the most suitable instruments for their assessment in specific populations, are currently under global review [3]. Accordingly, data collection tools must be carefully designed to evaluate self-concept, self-perception, physical activity, and lifestyle. Among adolescents, the Rosenberg Self-Esteem Scale (RSES) [45,46], the General Health Questionnaire-12 (GHQ-12) [47,48], the Physical Activity Questionnaire for Adolescents (PAQ-A) [33,49], and the Health Behaviour in School-aged Children (HBSC) [50,51] are recognized as the most appropriate instruments. Attention is also given to positive development, which fosters the acquisition of competencies, values, and skills [8,52,53,54]. Thus, adolescence represents a critical period for addressing health behaviors and psychological well-being, as habits formed during this stage often persist into adulthood [5,37,55,56,57].

Developing studies that provide valid tools for interventions aimed at promotion, prevention, treatment, rehabilitation, and education of adolescents and their environments is essential [30,58]. In line with this, the research team conducted a systematic review prior to the present study to evaluate self-concept, self-perception, physical activity, and lifestyle among adolescents internationally using these instruments [59]. The review revealed that men are more prone to life-threatening health problems, whereas women are more affected by disabling chronic conditions, resulting in higher negative self-perception among females. Mental health disorders, including depression and anxiety, are also more prevalent in women. These patterns appear consistent across cultures, despite expected improvements in educational systems in developed countries. The review emphasized the importance of promoting positive development in adolescents, particularly among females, who show the highest prevalence of psychiatric conditions worldwide. Regarding physical activity, men are generally more active, with younger Spaniards standing out. Additionally, the use of toxic substances is more common in these populations, and Spanish students display similar harmful behaviors [59].

Understanding adolescents’ emotions and behaviors is essential for fostering a healthier future adult population. Promoting self-care and healthy lifestyles from an early age helps establish enduring habits that can persist into adulthood. This research has practical implications, particularly for future health professionals, who are expected to serve as role models for healthy behaviors. Evaluating whether these students engage in self-care is crucial for developing effective strategies to support adolescent health, including potential adjustments to teaching methods. Furthermore, instilling healthy lifestyle practices in youth contributes to cultivating a more active, productive, and resilient workforce in the long term. From a public health perspective, encouraging healthy habits among adolescents may help reduce hospital admissions for chronic non-communicable diseases, thereby alleviating economic burdens.

### 1.3. Aims

The aim of this study is to evaluate self-concept, self-perception, engagement in physical activity, and lifestyle habits among undergraduate Health Sciences students, specifically those enrolled in the Psychology, Medicine, and Nursing programs at the University of La Laguna (ULL), Tenerife, Spain.

To achieve this aim, the study has two specific objectives: first, to analyze responses from the Rosenberg Self-Esteem Scale (RSES), General Health Questionnaire-12 (GHQ-12), Physical Activity Questionnaire for Adolescents (PAQ-A), and Health Behaviour in School-aged Children (HBSC) among Health Sciences students at ULL; and second, to compare results across the three academic programs—Psychology, Medicine, and Nursing—in order to identify intergroup differences and patterns reflecting the influence of academic environment and demographic factors on students’ well-being and health-related behaviors.

## 2. Materials and Methods

### 2.1. Design and Procedure

This was a descriptive, observational, cross-sectional study with analytical components. The Strengthening the Reporting of Observational Studies in Epidemiology (STROBE) [60] guidelines for this type of study were followed. First-year undergraduate students of Psychology, Medicine, and Nursing at the University of La Laguna (ULL), Tenerife (Spain), were invited to participate.

Data collection was carried online out during four days in February 2019 at 4 centers of the University of La Laguna, Tenerife (Spain): the School of Psychology; the School of Medicine; the Nursing School of the Canary Islands University Hospital; and the School of Nursing of the Nuestra Señora de Candelaria University Hospital.

### 2.2. Population and Sample

**Figure 1:**
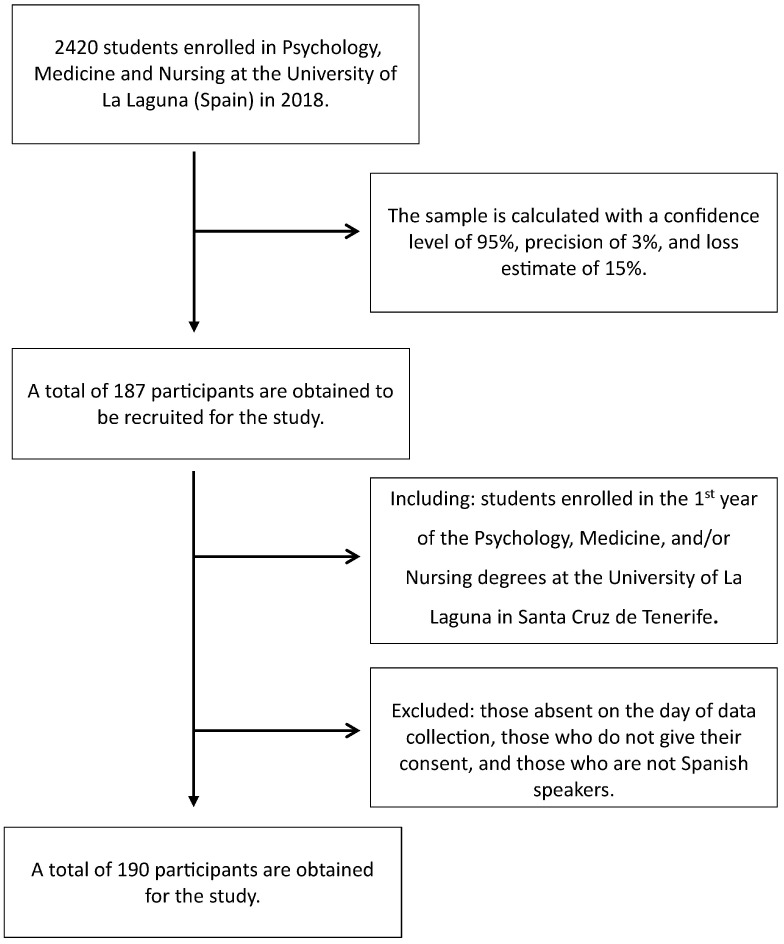
Flow chart showing the number of adolescents included in the analysis after applying the exclusion criteria.

The total population enrolled across the different study centers, according to data from the University of La Laguna, was 2420 in 2018 [61]. The sample size, calculated for a 95% confidence level, a 3% margin of error, and an estimated 15% attrition rate, was 187 participants, stratified by study center. The final sample comprised 190 participants (Figure 1). Non-probabilistic convenience sampling was employed, as all first-year students enrolled in the Psychology, Medicine, and Nursing programs who met the established inclusion criteria were invited to participate. This sampling method allowed the study to achieve the required sample size.

### 2.3. Inclusion Criteria

Adolescents and young adults in their 1st year of Psychology, Medicine, and Nursing studies at the University of La Laguna, Santa Cruz de Tenerife, were included.

### 2.4. Exclusion Criteria

Students who were not present on the day of data collection, those who did not give their consent, and those who were not Spanish speakers were excluded.

### 2.5. Variables

Demographic variables included age, sex, body mass index (BMI), and study program. In addition, self-concept, self-perception, physical activity, and lifestyle were included as key study variables. Data for these variables were collected using validated questionnaires [3], specifically the Rosenberg Self-Esteem Scale (RSES) [45,46], the General Health Questionnaire-12 (GHQ-12) [47,48], the Physical Activity Questionnaire for Adolescents (PAQ-A) [33,49], and the Health Behaviour in School-aged Children (HBSC) [50,51].

### 2.6. Instruments

RSES [45,46]: This is a two-dimensional scale assessing positive self-esteem (items 1, 3, 4, 7, and 10) and negative self-esteem (items 2, 5, 6, 8, and 9). A 4-point Likert- type scale is used, ranging from 1 (strongly disagree) to 4 (strongly agree). Negative self-esteem items are rated inversely. The authors did not establish cut-off points, although the following were considered:

- A score of 30 to 40 points: High self-esteem. This is considered normal self-esteem.
- A score of 26 to 29 points: Medium self-esteem. There are no serious self-esteem problems, but self-esteem should be improved.
- Less than 25 points: Low self-esteem. There are significant self-esteem problems.

GHQ-12 [47,48]: This explores depression, anxiety, social inadequacy, and hypochondriasis. It consists of 6 positive questions (items 1, 3, 4, 7, and 8) and 6 negative questions (items 2, 5, 6, 9, 10, and 11). It is assessed on a 4-point Likert scale. The higher the score, the greater the degree of emotional symptomatology. Scores of 12 or higher indicate the possibility of emotional disturbance.

PAQ-A [33,49]: This assesses physical activity in the last 7 days. It allows for the evaluation of when adolescents are most active. It consists of 9 questions and uses a 5-point Likert scale. The first 8 questions establish a gradation of the young person’s level of physical activity. The last question determines whether the adolescent is ill or not.

HBSC [50,51]: This explores several variables: sociodemographic characteristics, food and diet, oral hygiene, hours of sleep, physical activity and sedentary behaviors, risk consumption, sexual behavior, injuries, family context, peers and leisure time, school context, neighborhood, health and psychological adjustment, and socio-economic inequalities. The HBSC does not provide a total score that indicates whether the adolescent has an adequate lifestyle. In the studies conducted so far, only descriptive findings found at the international level have been observed. It should be noted that the HBSC study has a total of 19 sub-sections. In this research, those sub-sections required for the assessment of the lifestyle of late adolescents were included. For these reasons, the research team proposed a scoring method by sub-sections and an overall score:

- Food: Questions 1–4. Scores from 0 to 2 indicate a poor diet; 3 to 5 points reflect a fair diet; and scores from 6 to 8 indicate a good diet. Questions 5 and 6 were not considered as they were not significant for the adolescents’ eating habits.
- Body image: Questions 7–10. Scores below 2 indicate a bad body image; scores between 3 and 5 reflect a fair body image; and scores above 6 show a good body image.
- Sleep: Questions 11–14. Scores below 2 indicate that the person did not get enough sleep; scores between 3 and 5 points indicate they got a fair amount of sleep; and scores above 6 points indicate they got enough sleep.
- Violence: Questions 15–19. Scores below 4 indicate a high level of violence; scores between 5 and 7 points show that the level of violence is intermediate; and scores above 8 reflect that the person does not engage in or suffer violent acts.
- Positive health: Questions 20–24, 38, and 39. Scores below 4 indicate poor health; scores between 5 and 9 reflect fairly positive health; and scores above 10 indicate good positive health.
- Total HBSC score: This is obtained by adding the total scores of the previous sections. Thus, scores below 4 refer to a poor lifestyle; scores between 5 and 7 to a fair lifestyle; and scores above 8 to an adequate or healthy lifestyle.

Questions 25 to 37 of the survey were related to the Orientation to Life Questionnaire (OLQ-13) or Sense of Coherence (SOC-13) Scale [62,63,64]. This scale measures the sense of internal coherence related to coping with traumatic situations. It is a predictor of perceived and objective health. It is made up of 13 questions, of which items 25, 26, 27, 27, 31, and 34 are formulated in a negative sense, so their value needs to be inverted for the analysis. The SOC-13 scale has three subscales, which can be analyzed according to the following:

- Comprehensibility: This refers to the ability to understand other people and to control one’s thoughts and emotions. It allows for an adequate management of relationships with one’s social and interpersonal environment. It is assessed through questions 26, 30, 32, 33, and 35.
- Manageability: This refers to the degree of personal understanding of the available resources, under one’s own control or under the control of others, to cope with the demands of the environment. It is assessed through questions 27, 29, 34, and 37.
- Meaningfulness: This refers to the motivational component whereby demands are seen as challenges. It refers to questions 25, 28, 28, 31, and 36.
- The sum of each question would give a total score ranging from 13 to 91 points.

In the present study, the internal consistency of the questionnaires was assessed using Cronbach’s alpha coefficient. The following values were obtained in our sample: Rosenberg Self-Esteem Scale (RSES), α = 0.878; General Health Questionnaire-12 (GHQ-12), α = 0.234 for the total scale, with reliability improving after factor analysis in the subscales of self-worth perception (α = 0.732), confidence (α = 0.700), and concern (α = 0.476); Physical Activity Questionnaire for Adolescents (PAQ-A), α = 0.874; Health Behaviour in School-aged Children (HBSC), α = 0.887; and Sense of Coherence (SOC-13), α = 0.840, with subscale values of Comprehensibility α = 0.669, Manageability α = 0.539, and Meaningfulness α = 0.645. These results demonstrate adequate reliability for most of the instruments and subscales, except for the total GHQ-12, which justifies the factor analysis conducted.

### 2.7. Statistical Analysis

Statistical analyses were performed using IBM SPSS 22.0. Descriptive statistics were calculated using measures of central tendency (means) and percentages, without reporting confidence intervals or *p*-values, as these indicators are not appropriate for purely descriptive purposes and could lead to misinterpretation. Inferential statistics were applied to examine associations and group differences, using appropriate tests such as Pearson correlations, Student’s *t*-test, Kruskal–Wallis test, and post hoc comparisons, with their corresponding *p*-values reported. This approach maintained methodological consistency between the descriptive characterization of the sample and the inferential evaluation of specific hypotheses.

Although the instruments had been validated in previous studies, reliability was assessed in the present study using Cronbach’s alpha. Correlation analyses were also conducted to explore potential relationships between variables. As detailed in the results, factor analysis was performed on questionnaires exhibiting low reliability to identify underlying factors. A maximum likelihood method with Varimax rotation was applied, provided that Bartlett’s test of sphericity and the Kaiser–Meyer–Olkin (KMO) measure of sampling adequacy indicated suitability. Once factors, conceptualized as subscales, were identified, their reliability was evaluated using Cronbach’s alpha. The Kolmogorov–Smirnov test was applied to determine whether the data followed a normal distribution.

This study hypothesized that first-year students in Psychology, Medicine, and Nursing would demonstrate positive self-concept and self-perception, engage in physical activity, and maintain healthy lifestyle habits. Hypothesis testing was conducted to identify differences between groups based on sociodemographic variables, with the aim of determining whether sex, age, field of study, or socio-cultural level influenced these outcomes.

### 2.8. Ethical Aspects

All participants provided written informed consent prior to data collection. The study adhered to the ethical principles outlined in the Declaration of Helsinki for research involving human subjects. Ethical approval was granted by the University of La Laguna (ENF 19/43). Data confidentiality was maintained throughout both collection and analysis. All adolescents who participated had previously completed the written informed consent form, ensuring voluntary and confidential participation.

## 3. Results

### 3.1. Description of the Sample

The sample consisted of 190 participants, of whom 80% were women and 20% were men, with a median age of 19 years. The sample size ensured representativeness with a 95% confidence interval and a precision of 3%. Participants were enrolled in undergraduate programs in Psychology (34.2%), Medicine (18.4%), and Nursing (47.4%) at the University of La Laguna. Overall, a general tendency toward overweight was observed. Among male students, those in Nursing exhibited the highest levels of overweight. For female students, the highest levels of overweight were observed in the Psychology program, followed by Nursing. It is also noteworthy that underweight students were predominantly enrolled in Psychology, regardless of age. Detailed results are presented in Table 1.

### 3.2. Descriptive and Factor Analysis of the Questionnaires

Table 2 presents the results according to age, study program, and sex. As for the reliability of the responses to each questionnaire, the following Cronbach’s α values were obtained: 0.878 for the RSES, 0.234 for the GHQ-12, 0.874 for the PAQ-A, 0.887 for the HBSC, and 0.840 for the SOC-13. The results are expressed in Table 3.

In terms of self-esteem, assessed using the Rosenberg Self-Esteem Scale (RSES), most students exhibited medium to high levels. Male Nursing students had the lowest scores (below 25 points), whereas young male Psychology students (aged 19–24) showed particularly high self-esteem, with nearly 100% scoring at elevated levels. Female students generally reported moderate self-esteem, though older women (>25 years) tended to have lower scores. Overall, 50% of the sample scored above 30 points, indicating high self-esteem, 26.3% scored between 26 and 29 points (medium self-esteem), and 23.7% scored below 25 points (low self-esteem). Notably, a substantial proportion of students reported negative self-perceptions, with 34.2% feeling they were not good at anything, 40.5% feeling useless, and 61.6% believing they did not respect themselves.

Mental health, assessed with the GHQ-12, indicated a high prevalence of emotional disturbances, with 87.4% of students scoring above 12, suggesting notable psychological discomfort. Specifically, 46.3% reported feeling overwhelmed and stressed, 47.3% felt unable to overcome difficulties, 42.6% had lost self-confidence, and 60% perceived themselves as worthless. Analysis of the GHQ-12 subscales revealed that male Psychology students scored highest on the confidence subscale (above 7 points), women in Medicine scored lowest on the perception of self-value subscale (above 11 points), and female students across programs exhibited elevated concern scores (above 9 points), indicating persistent worry. Due to low reliability of the total GHQ-12, a factor analysis was conducted. Sampling adequacy was confirmed via Bartlett’s test of sphericity and the Kaiser–Meyer–Olkin test. Maximum likelihood analysis with Varimax rotation identified three subscales—perception of self-value (questions 1, 3, 4, 8, 12), confidence (questions 9, 10, 11), and concern (questions 2, 5, 6, 7)—which demonstrated higher reliability than the overall questionnaire.

Physical activity, assessed with the PAQ-A, was predominantly moderate across all groups, with vigorous activity less common, particularly among women and Medicine students. Female students also tended to be the most sedentary, scoring below 2 points on the PAQ-A. Walking was the most frequently reported activity, with 27.9% of respondents performing it more than four times per week, followed by running and muscle-building exercises. Most students remained seated before and after lunch (87.4%), and nearly half (49.5%) did not engage in exercise until after 6 p.m., with only 49.4% doing sport more than twice a week in the late afternoon. Weekend activity patterns were similar, with 37.4% of students devoting most of their free time to low-effort activities, and Sunday showing the lowest levels of physical activity. Overall, 20% of students were sedentary, 65.3% engaged in moderate activity, and 14.7% reached vigorous activity levels.

The assessment of health behaviors through the HBSC revealed that students generally perceived their lifestyle as fair or good, with 8.9% reporting a poor lifestyle, 58.9% a fair one, and 32.1% a good one. Eating habits were mostly fair, although young men in Psychology and Medicine exhibited poorer dietary patterns, including frequent consumption of sweets, crisps, and soft drinks. Despite this, the majority of students maintained a positive perception of their body image: 62.1% were satisfied with their appearance, although 31.1% felt they should lose weight and 43.6% considered their weight slightly or excessively high.

Sleep patterns indicated insufficient rest, as over half of the students (52.2%) went to bed after midnight on university days and 70.5% woke between 6 and 7 a.m., resulting in less than eight hours of sleep. Emotional well-being showed that many students experienced frustration or impatience: 51.5% felt upset when things did not go their way, 25.8% reported outbursts of anger, 52.1% admitted to impatience, and 38.9% struggled to wait for what they wanted. At the same time, social stressors such as perceived violence or exclusion were relatively low, with 9.4% reporting being insulted or laughed at, 16.3% feeling ignored, and 12.7% indicating that false rumors had been spread about them.

Overall, despite areas of concern in diet, sleep, and emotional regulation, most students rated their health positively, with 71.1% describing it as good or excellent, reflecting a generally adequate self-perception of health and well-being among the sample.

The Sense of Coherence (SOC-13) questionnaire indicated that most students experienced fair to good levels of positive health and coherence. Its subcomponents—Comprehensibility, Manageability, and Meaningfulness—were extracted using questions 25 to 37 of the HBSC and assessed via maximum likelihood analysis, with Bartlett’s test of sphericity and the Kaiser–Meyer–Olkin test confirming the adequacy of the sample. Reliability, evaluated with Cronbach’s alpha, was 0.840 for SOC-13, 0.669 for Comprehensibility, 0.539 for Manageability, and 0.645 for Meaningfulness. Based on a classification of bad, fair, or good, 80.5% of respondents scored at a fair level on SOC-13 (9–17 points), while nearly all participants achieved positive scores on the subscales, reflecting a strong life orientation and capacity to cope with challenging situations.

In summary, self-esteem and sense of coherence were relatively high across the sample, whereas emotional distress was highly prevalent, potentially reflecting academic stress or other psychosocial factors associated with university life. Moderate physical activity was common, but vigorous exercise was limited. Health and lifestyle behaviors were generally fair, with specific areas for improvement in eating habits and sleep, particularly among young men. Finally, perceptions of safety and coherence were positive, indicating resilience and a sense of security among most students.

### 3.3. Correlations Between Questionnaires

For the correlation analysis, the questionnaires, their mean scores, the factors identified in the previous section, and the derived variables were included. Table 4 summarizes the results, highlighting medium-to-large correlations between questionnaire factors and primary outcomes. The Rosenberg Self-Esteem Scale (RSES) showed highly significant positive correlations with perceived self-value (*r* = 0.500, *p* < 0.001) and confidence (*r* = 0.556, *p* < 0.001) while correlating negatively with concern (*r* = −0.280, *p* < 0.001). RSES also correlated strongly with the total Sense of Coherence (SOC-13) score (*r* = 0.581, *p* < 0.001) and its subscales, indicating that higher self-esteem is associated with a stronger sense of coherence.

The General Health Questionnaire (GHQ-12) exhibited significant negative correlations with both the HBSC lifestyle questionnaire (*r* = −0.153, *p* = 0.063, marginally significant) and SOC-13 (*r* = −0.188, *p* = 0.009), suggesting that higher emotional distress is associated with poorer lifestyle and lower coherence. Additionally, GHQ-12 correlated positively with concern (*r* = 0.649, *p* < 0.001).

The Physical Activity Questionnaire for Adolescents (PAQ-A) correlated positively with positive health (*r* = 0.175, *p* = 0.016) and negatively with perceived violence (*r* = −0.162, *p* = 0.025), reflecting the benefits of physical activity on well-being and social perception.

The HBSC lifestyle questionnaire showed significant positive correlations with eating habits (*r* = 0.625, *p* < 0.001), body image (*r* = 0.563, *p* < 0.001), sleep quality (*r* = 0.598, *p* < 0.001), positive health (*r* = 0.634, *p* < 0.001), and SOC-13 (*r* = 0.471, *p* < 0.001), reinforcing the internal consistency of the lifestyle construct and its close association with health and coherence measures.

Overall, these patterns indicate that higher self-esteem, positive health behaviors, and physical activity are interrelated and inversely associated with emotional distress and concern, supporting the convergent validity of the instruments used in the study.

### 3.4. Hypothesis Testing

Table 5 summarizes the results of hypothesis testing according to age, sex, and study program. For age, participants were divided into two groups, younger than 19 years (<19) and 19 years or older (≥19), as the small number of participants over 25 years (*n* = 2) precluded independent analysis. Analyses of the questionnaires and their subscales using Student’s *t*-test and the Kruskal–Wallis test revealed no significant differences across age groups (*p* > 0.05).

When comparing study programs, the Kruskal–Wallis test was applied due to the non-normal distribution of some variables. Significant differences were observed for perceived self-value (GHQ-12; *χ*^2^ = 12.4, *p* < 0.001), physical activity (PAQ-A; *χ*^2^ = 9.6, *p* = 0.003), and sleep quality (HBSC; *χ*^2^ = 14.2, *p* < 0.001). Post hoc analyses indicated that Psychology students reported lower perceived self-value than both Nursing and Medicine students (*p* < 0.01), Medicine students had higher PAQ-A scores than Nursing students (*p* < 0.05), and sleep quality differed significantly between Medicine and Nursing students (*p* < 0.01). Other variables showed no statistically significant differences, suggesting insufficient evidence to reject the null hypothesis of equality.

Sex-based comparisons revealed that males scored significantly higher than females on the SOC-13 subscale of Comprehensibility (*t* = 0.90, *p* = 0.04), while no differences were observed for RSES, GHQ-12, or HBSC scores (*p* > 0.05). Scores for the Manageability and Meaningfulness subscales of SOC-13 were uniform across participants (median = 2.0), precluding statistical comparison.

Overall, these findings indicate that age did not significantly influence questionnaire outcomes, while study program and sex contributed to specific differences in perceived self-value, physical activity, sleep quality, and the sense of coherence subscale of Comprehensibility.

## 4. Discussion

Our findings suggest a possible discrepancy between the knowledge they acquire and their own health behaviors. Despite being future healthcare professionals, many display low self-esteem and poor self-concept—particularly among men—while women tend to report low levels of self-confidence. In this sample, confidence appeared lower in later university years than among secondary students, which may indicate that academic progression is not consistently associated with gains in personal well-being. Although female students reduce sedentary behavior over time, by their fourth year they paradoxically report the poorest lifestyles. In contrast, male students become increasingly sedentary throughout their studies.

This disconnect may point to limitations in current health promotion approaches, which may be insufficient to support behavioral change in some contexts. Rethinking how self-care is addressed during training, emphasizing evidence-based actions, effective pedagogical tools, and broader institutional involvement, may be warranted.

When comparing these findings with those of a previous systematic review [59], clear similarities emerge. Both studies highlight that women tend to present worse health conditions, reflected in higher BMI, poorer self-concept, and lower self-perception, while men generally report higher levels of physical activity. However, the role of cultural changes in shaping these lifestyles remains unclear. Prior studies have noted that Nursing students may neglect their own health behaviors, and our findings are consistent with this concern, particularly by identifying high consumption of unhealthy foods among female students in Psychology and Nursing programs.

Self-concept, as measured by the RSES, revealed that 62.9% of male Nursing students under the age of 24 scored below 25 points, indicating low self-esteem. A similar pattern was observed among women, particularly in Psychology, where 34.8% of 18-year-old students reported low self-esteem, followed by 32% in Medicine and 28.9% in Nursing within the 19–24 age group. Previous research has suggested that physical self-concept is closely linked to physical activity and that general self-concept tends to decline with age [9]. However, in the present study, the opposite trend emerged: older students showed higher self-concept scores. This finding suggests that self-esteem in university students may be shaped more by academic and social factors than by age alone. Indeed, research indicates that self-esteem is associated with academic engagement and emotional well-being, serving as a significant predictor of both participation and performance [65,66]. Such outcomes may reflect processes of maturational development and personal acceptance acquired during university years.

In the GHQ-12, male Psychology students reported the lowest levels of confidence (scores above 7 on the confidence subscale), with 75% of these cases corresponding to 18-year-olds. This was followed by Medicine students aged 25 and older, of whom 50% also showed low confidence. This result is particularly striking, as previous studies suggest that self-perception and self-confidence generally improve as students progress through their university courses [59,67]. Among women, 42.2% of Nursing students presented low confidence, also reflected in scores above 7 on the same subscale.

Regarding the perception of value, 100% of male Psychology students in late adolescence scored within the fair range (6–10 points), whereas female students of the same age showed more positive results, with 70% scoring good (below 5 points) and only 30% scoring fair. A similar pattern was observed among Nursing students, while in Medicine, it was women who reported the poorest perception of value, with scores above 11 points. Additionally, Psychology students aged 18–24 tended to show fair levels of concern, which appeared to worsen with age, although the lowest results were found among Medicine students in the same age group.

These findings align with the previous literature, which emphasizes the relationship between self-perception, mental health, and lifestyle. For instance, Luna-Alfaro et al. [5] reported that greater body image perception is associated with poorer eating habits, and vice versa. Similarly, differences in mental health indicators among university students have been shown to vary across gender, age, and academic discipline [68], while academic and social environments may further shape students’ self-perception and confidence [69,70].

With respect to physical activity, the findings indicate that women were the most sedentary group. The PAQ-A revealed an overall average level of activity (scores below 2), with Psychology students being the least active (scores between 3 and 4). These results are consistent with previous studies reporting significant associations between sedentary lifestyles and psychological distress, including depression, dissatisfaction with life, and reduced happiness in adolescents. Conversely, physical activity has been shown to improve self-esteem, self-concept, and self-perception [28,29]. Nevertheless, much of the literature suggests that men are generally more inclined toward physical activity [31]. Moreno et al. further argue that physical self-concept influences individuals’ intention to be active, as it tends to be positively shaped by regular exercise [32]. In contrast, the present study identified a negative correlation between these two variables.

Previous evidence highlights that university students who engage in regular physical activity are less likely to experience depression and anxiety, even under stressful conditions such as COVID-19 restrictions [71]. Conversely, sedentary behavior has been linked to adverse effects on mental health in this population [72]. Taken together, these findings suggest that adolescents may not be significantly affected by body image—either due to personal acceptance or to a lack of engagement in modifying lifestyle habits.

In terms of lifestyle, as assessed through the HBSC, women reported the best eating habits (scores above 6), particularly among Medicine (36%) and Nursing students (24.4%). However, this contrasted with body image perception, where young men obtained the highest scores (above 6). Notably, 100% of Psychology students aged 19–24 years reported positive body image, followed by 60% of Nursing and Medicine students. Sleep habits were generally poor across both sexes (scores below 5), although females showed comparatively better results. Despite overall low rates of violence, certain groups reported experiencing or engaging in violent acts, including 14.3% of men and women in Nursing and 8.7% of women in Psychology (scores below 7). Regarding positive health, men in Medicine scored highest (above 8), followed by women in Nursing.

These findings are consistent with previous studies. For example, Laguado-Jaimes et al. reported high levels of overweight and obesity among Nursing students, alongside unhealthy behaviors such as inadequate food intake and sedentary lifestyles [42]. Similarly, dietary habits in university students are closely linked to perceived health and overall well-being [73]. Furthermore, sleep quality in this population has been shown to be influenced by both dietary patterns and general health behaviors [74].

Among women aged 19–24, SOC-13 scores were lower in Medicine (8%), Nursing (4.4%), and Psychology (3.3%), defined as scores below 8 points. However, when analyzed by subscales, more favorable outcomes were observed. The only significant sex-based difference appeared in the Comprehensibility dimension, where males achieved higher scores (above 8 points). Previous studies have confirmed that the SOC-13 demonstrates acceptable psychometric properties, including internal consistency and factorial structure, across diverse populations such as university students [75]. Moreover, sense of coherence in this population has been shown to be closely associated with mental health and overall well-being [76].

### 4.1. Limitations

Several limitations of this study should be acknowledged. First, the use of convenience sampling may have introduced selection bias, as some students were not surveyed due to absence on the day of data collection. Furthermore, the cross-sectional design prevents establishing causal relationships and limits the ability to examine how the variables may evolve over time. These methodological choices may reduce the generalizability and representativeness of the findings; future studies could strengthen these aspects with more robust sampling procedures and larger samples.

Second, although data collection was completed within four consecutive days in February 2019, the analysis, writing, and editorial process coincided partly with the COVID-19 pandemic. This context may have influenced timelines and should also be considered as a potential factor affecting students’ mental and physical health. Future studies should investigate whether the pandemic has altered health behaviors and lifestyles in ways that differ from those observed in this study, which was conducted under pre-pandemic conditions.

Third, the validity and reliability of the findings are conditioned by the instruments used (RSES, GHQ-12, PAQ-A, HBSC, SOC-13). The PAQ-A, although widely used, was originally designed for adolescents and focuses on physical activity in the past seven days, which may not fully reflect the activity patterns of college students with different responsibilities. Similarly, the HBSC questionnaire, tailored primarily for school-aged children (11–15 years), may not capture the more complex health behaviors of older students.

Regarding the GHQ-12, internal consistency for the total scale in this sample was low (α = 0.234), which limits the reliability of the results. To address this, an exploratory factor analysis was conducted, identifying three subscales (perceived value, confidence, and concern), two of which showed acceptable reliability. Although these subscales are not part of the original validation, their use has been described in the literature as a strategy to enhance interpretability in specific contexts [77,78,79]. Nevertheless, results derived from the GHQ-12 should be interpreted with caution, and further research is recommended to evaluate its factorial structure in similar university populations.

Finally, regarding the classifications used in the HBSC and SOC-13 instruments, it should be clarified that these were not arbitrary cut-off points but methodological adaptations based on the existing literature [80,81,82] and were agreed upon by the research team. These adaptations allowed for a more operational interpretation of the results without altering the original scores, and they were applied uniformly across the entire sample.

### 4.2. Practical Implications

The findings of this study highlight the urgent need to strengthen strategies for promoting physical activity and healthy lifestyles among health sciences students. Despite their academic training, students often display a concerning discrepancy between the knowledge they acquire and their own health behaviors. Moreover, confidence and self-perception tend to decrease as they progress through their studies, suggesting that current academic training does not sufficiently reinforce personal well-being [83]. Addressing these gaps is essential, since habits established during this stage are likely to persist into adulthood, shaping long-term health outcomes.

Given that the participants were enrolled in Psychology, Nursing, and Medicine programs, their future professional role in transmitting healthy lifestyle practices to the general population reinforces the importance of early intervention. Developing a positive self-concept and self-perception during adolescence and early adulthood contributes to emotional stability, reduces the risk of chronic non-communicable diseases, and fosters a healthier population, with implications for both healthcare systems and broader socioeconomic outcomes.

From a practical perspective, this research suggests the need for an integrated approach to health sciences education, one that goes beyond teaching methodologies to include curriculum reform. Specifically, it is recommended that educators

- Implement participatory and experiential strategies that actively engage students in health promotion and self-care, linking theory with real-world practice in both academic and community contexts.
- Introduce structured health promotion programs from the first year of university, emphasizing both physical activity and psychological well-being.
- Incorporate mandatory, cross-curricular content on health education, stress management, and self-care throughout the academic programs.
- Expand opportunities for physical activity within the university setting, not only to improve physical performance but also to enhance psychological well-being, group cohesion, and reduce sedentary behaviors.
- Create institutional spaces and resources that support sports practice, mental health, and self-care.
- Integrate continuous training on healthy habits, emotional management, and risk prevention into core courses, ensuring these topics are addressed consistently rather than as isolated activities.

Additionally, it is important to continue examining the evolution of self-concept, self-perception, physical activity, and lifestyle throughout students’ university years. Comparative studies between first- and fourth-year students could provide valuable insight into whether increased knowledge of healthy lifestyle habits translates into improved personal practices. For instance, recent findings published in 2023 confirm significant improvements in self-esteem, sense of coherence, and lifestyle among fourth-year Nursing students compared to their first-year counterparts, underscoring the role of university as a stage for consolidating these dimensions [83]. However, longitudinal designs face inherent challenges, as extended follow-up periods may result in attrition once students graduate or leave the institution.

In summary, universities should prioritize strategies that not only enhance academic knowledge but also foster the adoption of coherent, healthy behaviors among health sciences students, ensuring they are well-prepared to act as credible promoters of health in their professional careers.

## 5. Conclusions

Health Sciences students in this study demonstrated low self-concept, emotional distress, sedentary behaviors, and lifestyles in need of improvement. These findings are particularly noteworthy given that, as future health professionals, students are expected to recognize health risks and promote healthy habits. Despite having prior knowledge of healthy lifestyles and exposure to health promotion education, many first-year students did not consistently translate this knowledge into coherent behaviors.

This emphasizes the importance of reinforcing educational and curricular strategies from the beginning of university training, a period when students are still adapting to academic and personal changes. Such strategies should focus on transforming theoretical knowledge into sustainable health habits and supporting students throughout their academic journey. The persistence of less favorable outcomes in this population suggests that the problem is not solely due to a lack of prior exposure but also reflects the need for more targeted, context-specific educational approaches that foster consistent and sustainable behaviors.

In addition, differences were observed across subgroups. Male Nursing students and female Psychology students reported the lowest levels of self-concept compared to their peers, while female Nursing students expressed lower confidence relative to Medicine and Psychology students. These outcomes may be influenced by developmental factors, including the personal insecurities characteristic of late adolescence. Monitoring whether these variables evolve with the acquisition of knowledge and personal growth is essential for designing interventions that effectively strengthen students’ well-being and professional competencies.

## Figures and Tables

**Table 1:** Sociodemographic descriptive results.

	Males						Females						Total
	<19 Years			≥19 Years			<19 Years			≥19 Years			
	Under	Normal	Over	Under	Normal	Over	Under	Normal	Over	Under	Normal	Over	
	Weight	Weight	Weight	Weight	Weight	Weight	Weight	Weight	Weight	Weight	Weight	Weight	
Psychology	0	4	0	0	3	2	7	11	5	5	16	12	
	0%	44.4%	0%	0%	16.7%	22.2%	100%	47.8%	62.5%	55.6%	18.8%	60.0%	
Total	*n* = 9; 23.7%						*n* = 56; 36.8%						*n* = 65; 34.2%
Nursing	0	5	2	0	10	5	0	12	3	3	43	7	
	0%	55.6%	100%	0%	55.5%	55.5%	0%	52.2%	37.5%	33.3%	50.6%	35.0%	
Total	*n* = 22; 57.9%						*n* = 68; 44.7%						*n* = 90; 47.4%
Medicine	0	0	0	0	5	2	0	0	0	1	26	1	
	0%	0%	0%	0%	27.8%	22.2%	0%	0%	0%	11.1%	30.6%	5.0%	
Total	*n* = 7; 18.4%						*n* = 28; 18.4%						*n* = 35; 18.4%

**Table 2:** Differences by sex, study program, and age.

		Males								Females							
		Psychology			Nursing			Medicine		Psychology			Nursing			Medicine	
		18	19–24	>25	18	19–24	>25	19–24	>25	18	19–24	>25	18	19–24	>25	19–24	>25
RSES	High							60%	100%	34.8%	70%	66.7%					
								*n* = 5; 71.4%		*n* = 31; 55.4%							
	Medium	25%	100%	50%	14.3%	30%	20%						33.3%	26.7%	50%	32%	
		*n* = 4; 44.4%			*n* = 5; 22.7%								*n* = 21; 30.9%			*n* = 8; 28.6%	
	Low				42.9%	20%							6.7%	28.9%	25%	32%	33.3%
					*n* = 5; 22.7%								*n* = 16; 23.5%			*n* = 9; 32.1%	
GHQ-12	Normal															4%	
	Emotional disorder	100%			100%			100%		100%			100%			96%	100%
Confidence	Good							60%	50%							56%	
								*n* = 4; 57.1%								*n* = 14; 50%	
	Fair	0%	100%	75%	42.9%	40%	80%			26.1%	46.7%	33.3%	46.7%	15.6%	62.5%		
		*n* = 4; 44.4%			*n* = 11; 50%					*n* = 21; 37.5%			*n* = 19; 27.9%				
	Bad				28.6%	20%	20%			30.4%	20%	33.3%	33.3%	42.2%	0%		
					*n* = 5; 22.7%					*n* = 14; 25%			*n* = 24; 35.3%				
Perception	Good				57.1%	60%	80%			52.2%	70%	66.7%	80%	75.6%	62.5%		
					*n* = 14; 63.6%					*n* = 35; 62.5%			*n* = 51; 75%				
	Fair	0%	100%	25%				40%	0%							68%	66.7%
		*n* = 2; 22.2%						*n* = 2; 28.6%								*n* = 19; 67.9%	
	Bad							20%	0%							12%	0%
								*n* = 1; 14.3%								*n* = 3; 10.7%	
Concern	Good				14.3%	50%	20%	60%	50%				33.3%	33.3%	25%		
					*n* = 7; 31.8%			*n* = 4; 57.1%					*n* = 22; 32.4%				
	Fair	100%	100%	75%	71.4%	50%	80%			65.2%	60%	66.7%	53.3%	55.6%	75%	48%	33.3%
		*n* = 8; 88.9%			*n* = 14; 63.6%					*n* = 35; 62.5%			*n* = 39; 57.4%			*n* = 13; 46.4%	
	Bad									21.7%	16.7%	0%	13.3%	11.1%	0%	32%	0%
										*n* = 10; 17.9%			*n* = 7; 10.3%			*n* = 8; 28.6%	
PAQ-A	Sedentary				14.3%	10%	20%			34.8%	33.3%	0%	20%	13.3%	12.5%	16%	33.3%
					*n* = 3; 13.6%					*n* = 18; 31.1%			*n* = 10; 14.7%			*n* = 5; 17.9%	
	Moderate activity	75%	100%	50%	57.1%	50%	80%	100%	50%	52.2%	56.7%	100%	60%	80%	75%	56%	66.7%
		*n* = 6; 66.7%			*n* = 13; 59.1%			*n* = 6; 85.7%		*n* = 32; 57.1%			*n* = 51; 75%			*n* = 16; 57.1%	
	Vigorous activity				28.6%	40%	0%									28%	0%
					*n* = 6; 27.3%											*n* = 7; 25%	
HBSC	Fair	50%	100%	50%	85.7%	50%	60%	60%	50%	47.8%	56.7%	33.3%	60%	62.2%	87.5%	56%	66.7%
		*n* = 5; 55.6%			*n* = 14; 63.6%			*n* = 4; 57.1%		*n* = 29; 51.8%			*n* = 44; 64.7%			*n* = 16; 57.1%	
	Good				14.3%	50%	40%									40%	33.3%
					*n* = 8; 36.4%											*n* = 11; 39.3%	
Eating habits	Bad	75%	100%	25%				40%	0%								
		*n* = 5; 55.6%						*n* = 2; 28.6%									
	Fair				85.7%	80%	80%	40%	50%	52.2%	73.3%	66.7%	53.3%	60%	50%	56%	66.7%
					*n* = 18; 81.8%			*n* = 3; 42.9%		*n* = 36; 64.3%			*n* = 39; 57.4%			*n* = 16; 57.1%	
	Good				0%	20%	0%									36%	33.3%
					*n* = 2; 9.1%											*n* = 10; 35.7%	
Body image	Fair				42.9%	40%	20%						20%	48.9%	25%	44%	33.3%
					*n* = 8; 36.4%								*n* = 27; 39.7%			*n* = 12; 42.9%	
	Good	25%	100%	25%	57.1%	60%	80%	60%	50%	56.5%	56.7%	66.7%	80%	48.9%	75%	56%	66.7%
		*n* = 3; 33.3%			*n* = 14; 63.6%			*n* = 4; 57.1%		*n* = 32; 57.1%			*n* = 40; 58.8%			*n* = 16; 57.1%	
Sleep	Bad	75%	100%	50%	28.6%	40%	20%	40%	0%	65.2%	46.7%	0%	40%	53.3%	25%		
		*n* = 6; 66.7%			*n* = 7. 31.8%			*n* = 2; 28.6%		*n* = 29; 51.8%			*n* = 32; 47.1%				
	Fair				71.4%	40%	40%	60%	50%	21.7%	46.7%	66.7%	46.7%	42.2%	62.5%	68%	0%
					*n* = 11; 50%			*n* = 4; 57.1%		*n* = 21; 37.5%			*n* = 31; 45.6%			*n* = 17; 60.7%	
Violence	Bad												0%	2.2%	0%		
													*n* = 1; 1.5%				
	Fair												0%	2.2%	0%		
													*n* = 1; 1.5%				
	Good	100%	100%	100%	85.7%	100%	100%	100%	100%	91.3%	100%	100%	100%	95.6%	100%	100%	100%
		*n* = 9; 100%			*n* = 21; 95.5%			*n* = 7; 100%		*n* = 54; 96.4%			*n* = 66; 97.1%			*n* = 28; 100%	
Positive health	Fair	25%	100%	75%	71.4%	70%	20%	40%	50%	60.9%	50%	33.3%	60%	42.2%	75%	52%	66.7%
		*n* = 5; 55.6%			*n* = 13; 59.1%			*n* = 3; 42.9%		*n* = 30; 53.6%			*n* = 34; 50%			*n* = 15; 53.6%	
	Good				28.6%	30%	80%	60%	50%	21.7%	43.3%	66.7%	40%	46.7%	25.0%	32%	33.3%
					*n* = 9; 40.9%			*n* = 4; 57.1%		*n* = 20; 35.7%			*n* = 29; 42.6%			*n* = 9; 32.1%	
SOC-13	Fair	100%	100%	75%	100%	80%	100%	80%	50%	91.3%	80%	66.7%	80%	71.1%	100%	72%	100%
		*n* = 8; 88.9%			*n* = 20; 90.9%			*n* = 5; 71.4%		*n* = 47; 83.95			*n* = 52; 76.5%			*n* = 21; 75%	
Comprehensibility	Good	100%	100%	100%	100%	100%	100%	100%	100%	100%	96.7%	100%	93.3%	97.8%	100%	96%	100%
		*n* = 9; 100%			*n* = 22; 100%			*n* = 7; 100%		*n* = 55; 98.2%			*n* = 66; 97.1%			*n* = 27; 96.4%	
Manageability	Good	100%	100%	100%	100%	100%	100%	100%	100%	100%	100%	100%	100%	100%	100%	100%	100%
		*n* = 9; 100%			*n* = 22; 100%			*n* = 7; 100%		*n* = 56; 100%			*n* = 68; 100%			*n* = 28; 100%	
Meaningfulness	Good	100%	100%	100%	100%	100%	100%	100%	100%	100%	100%	100%	100%	100%	100%	100%	100%
		*n* = 9; 100%			*n* = 22; 100%			*n* = 7; 100%		*n* = 56; 100%			*n* = 68; 100%			*n* = 28; 100%	

**Table 3:** Descriptive results of the questionnaires.

Scales	Subscales	Evaluated Level	Response Percentage	α Cronbach	Bartlett’s Sphericity Test	Kaiser–Meyer–Olkin (KMO) Test	Chi-Squared
RSES		High self-esteem	50%	0.878			
		Medium self-esteem	26.3%				
		Low self-esteem	23.7%				
GHQ-12	Total	Emotional disorder	87.4%	0.234			
		Normal self-perception	12.6%				
	Perceived worth	Good	61.6%	0.732	sig: 0.000	0.807	40.432
		Fair	35.8%				
		Bad	2.6%				
	Confidence	Good	37.9%	0.7			
		Fair	35.8%				
		Bad	26.3%				
	Concern	Good	27.4%	0.476			
		Fair	58.9%				
		Bad	13.7%				
PAQ-A		Sedentary	20.0%	0.874			
		Moderate activity	65.3%				
		Vigorous activity	14.7%				
HBSC	Total	Bad	8.9%	0.887			
		Fair	58.9%				
		Good	32.1%				
	Eating habits	Bad	16.3%				
		Fair	60.5%				
		Good	23.2%				
	Body image	Bad	1.6%				
		Fair	41.1%				
		Good	57.4%				
	Sleep	Bad	42.6%				
		Fair	45.8%				
		Good	11.6%				
	Violence	Bad	0.5%				
		Fair	2.1%				
		Good	97.4%				
	Positive health	Bad	7.9%				
		Fair	52.6%				
		Good	39.5%				
SOC 13	Total	Bad	2.6%	0.840			
		Fair	80.5%				
		Good	16.8%				
	Comprehensibility	Bad	0%	0.669	sig: 0.000	0.799	37.623
		Fair	2.1%				
		Good	97.9%				
	Manageability	Bad	0%	0.539			
		Fair	0%				
		Good	100%				
	Meaningfulness	Bad	0%	0.645			
		Fair	0%				
		Good	100%				

**Table 4:** Correlations.

		RSES	GHQ12	Perceived Self-Value	Confidence	Concern	PAQ-A	HBSC	Eating Habits	Body Image	Sleep	Violence	Positive Health	SOC-13	Comprehensibility	Manageability	Meaningfulness
RSES	Pearson	1															
	Sig. (bil.)																
GHQ12	Pearson	−0.136	1														
	Sig. (bil.)	0.062															
Perceived self-value	Pearson	0.500 **	0.577 **	1													
	Sig. (bil.)	0.000	0.000														
Confidence	Pearson	0.556 **	0.141	0.488 **	1												
	Sig. (bil.)	0.000	0.052	0.000													
Concern	Pearson	0.265 **	0.649 **	0.292 **	−0.280 **	1											
	Sig. (bil.)	0.000	0.000	0.000	0.000												
PAQ-A	Pearson	−0.078	−0.102	−0.030	−0.035	−0.095	1										
	Sig. (bil.)	0.282	0.161	0.684	0.629	0.191											
HBSC	Pearson	0.406 **	−0.153 *	0.322 **	0.339 **	−0.243 **	0.118	1									
	Sig. (bil.)	0.000	0.063	0.000	0.000	0.001	0.105										
Eating habits	Pearson	0.151 *	−0.098	0.150 *	0.197 **	−0.233	0.133	0.625 **	1								
	Sig. (bil.)	0.037	0.178	0.038	0.007	0.001	0.067	0.000									
Body image	Pearson	0.393 **	−0.028	0.216 **	0.308 **	−0.085	0.066	0.563 **	0.206 **	1							
	Sig. (bil.)	0.000	0.699	0.003	0.000	0.245	0.366	0.000	0.004								
Sleep	Pearson	0.084	−0.051	−0.029	0.026	−0.080	0.075	0.598 **	0.260 **	0.141	1						
	Sig. (bil.)	0.251	0.483	0.694	0.722	0.271	0.307	0.000	0.000	0.052							
Violence	Pearson	−0.013	−0.040	−0.054	0.080	−0.099	−0.162 *	0.020	−0.021	−0.063	−0.047	1					
	Sig. (bil.)	0.855		0.457	0.272	0.174	0.025	0.788	0.774	0.387	0.522						
Positive health	Pearson	0.557 **	−0.272 **	0.492 **	0.465 **	−0.388 **	0.175 *	0.634 **	0.320 **	0.378 **	0.187 **	0.125	1				
	Sig. (bil.)	0.000	0.000	0.000	0.000	0.000	0.016	0.000	0.000	0.000	0.010	0.085					
SOC-13	Pearson	0.581 **	−0.188 **	0.429 **	0.451 **	−0.330 **	0.080	0.471 **	0.290 **	0.290 **	0.210 **	0.003	0.639 **	1			
	Sig. (bil.)	0.000	0.009	0.000	0.000	0.000	0.275	0.000	0.000	0.000	0.04	0.967	0.000				
Comprehensibility	Pearson	0.473 **	−0.171 *	0.323 **	0.341 **	−0.317 **	0.058	0.400 **	0.205 **	0.337 **	0.206 **	−0.069	0.513 **	0.848 **	1		
	Sig. (bil.)	0.000	0.018	0.000	0.000	0.000	0.429	0.000	0.004	0.000	0.004	0.341	0.000	0.000			
Manageability	Pearson	0.510 **	−0.118	0.326 **	0.359 **	−0.238 **	0.052	0.449 **	0.279 **	0.230 **	0.208 **	0.016	0.585 **	0.870 **	0.6430 **	1	
	Sig. (bil.)	0.000	0.104	0.000	0.000	0.000	0.474	0.000	0.000	0.001	0.004	0.822	0.000	0.000	0.000		
Meaningfulness	Pearson	0.464 **	−0.171 *	0.413 **	0.417 **	−0.253 **	0.082	0.366 **	0.272 **	0.207 **	0.133	0.079	0.543 **	0.761 **	0.421 **	0.568 **	1
	Sig. (bil.)	0.000	0.019	0.000	0.000	0.000	0.263	0.000	0.000	0.004	0.068	0.278	0.000	0.000	0.000	0.000	

** The correlation is significant at 0.01 level (bilateral). * The correlation is significant at 0.05 level (bilateral).

**Table 5:** Hypothesis testing.

	Age				Sex				Study Program
	Age	Sample	Mean	Student’s *t*	Sex	Sample	Mean	Student’s *t*	Kruskal–Wallis
RSES	<19	49	1.77	0.70	Male	38	1.57	0.18	0.57
	≥19	141	1.72	0.70	Female	152	1.77	0.16	
GHQ-12	<19	49	2.00	0.55	Male	38	2.00	0.61	0.10
	≥19	141	1.99	0.31	Female	152	1.99	0.31	
Confidence	<19	49	1.02	0.16	Male	38	0.94	0.58	0.06
	≥19	141	0.83	0.17	Female	152	0.86	0.57	
Perceived self-value	<19	49	0.34	0.34	Male	38	0.36	0.59	0.00
	≥19	141	0.43	0.30	Female	152	0.42	0.59	
Concern	<19	49	0.97	0.13	Male	38	0.71	0.09	0.12
	≥19	141	0.82	0.12	Female	152	0.90	0.05	
PAQ-A	<19	49	0.89	0.49	Male	38	1.07	0.12	0.03
	≥19	141	0.96	0.52	Female	152	0.91	0.12	
HBSC	<19	49	1.14	0.23	Male	38	1.28	0.50	0.23
	≥19	141	1.26	0.25	Female	152	1.21	0.49	
Eating habits	<19	49	1.00	0.37	Male	38	0.89	0.05	0.20
	≥19	141	1.09	0.40	Female	152	1.11	0.05	
Body image	<19	49	1.61	0.40	Male	38	1.55	0.94	0.69
	≥19	141	1.53	0.38	Female	152	1.55	0.94	
Sleep	<19	49	0.57	0.15	Male	38	0.73	0.62	0.00
	≥19	141	0.73	0.15	Female	152	0.67	0.63	
Violence	<19	49	1.93	0.23	Male	38	1.97	0.85	0.55
	≥19	141	1.97	0.29	Female	152	1.96	0.83	
Positive health	<19	49	1.24	0.34	Male	38	1.44	0.14	0.60
	≥19	141	1.34	0.34	Female	152	1.28	0.09	
SOC-13	<19	49	1.10	0.439	Male	38	1.13	0.86	0.87
	≥19	141	1.15	0.35	Female	152	1.14	0.84	
Comprehensibility	<19	49	1.97	0.97	Male	38	2.00	0.31	0.90
	≥19	141	1.97	0.97	Female	152	1.97	0.04	
Manageability	<19	49	2.00	-	Male	38	2.00	-	1
	≥19	141	2.00	-	Female	152	2.00	-	
Meaningfulness	<19	49	2.00	-	Male	38	2.00	-	1
	≥19	141	2.00	-	Female	152	2.00	-	

## Data Availability

The data of this study has not been deposited into a publicly available repository. This data is confidential and will be made available on reasonable request to the authors.

## Abbreviations

The following abbreviations are used in this manuscript:

BMI	Body Mass Index
GHQ-12	General Health Questionnaire-12 items
HBSC	Health Behaviour in School-aged Children
OLQ-13	Orientation to Life Questionnaire-13 items
PAQ-A	Physical Activity Questionnaire for Adolescents
RSES	Rosenberg Self-Esteem Scale
SOC-13	Sense of Coherence Scale-13 items
STROBE	Strengthening the Reporting of Observational Studies in Epidemiology
ULL	Universidad de La Laguna
WHO	World Health Organization
